# Cerebral malaria presenting as nonconvulsive status epilepticus: a case report

**DOI:** 10.1186/s12936-024-04908-z

**Published:** 2024-03-18

**Authors:** Xingguo Wu, Ningxiang Qin, Fahang Yi, Jing Wang, Xia Yan, Liang Wang

**Affiliations:** 1 Department of Neurology, The First Affiliated Hospital of Chongqing Medical University, Chongqing, China; 2 Jinsha County People’s Hospital, Guizhou, China

**Keywords:** Cerebral malaria, Nonconvulsive status epilepticus, Electroencephalogram

## Abstract

**Background:**

Malaria is an infectious malady caused by *Plasmodium* parasites, cerebral malaria standing out as one of its most severe complications. Clinical manifestation include elevated body temperature, loss of consciousness, and seizures. However, reports of cerebral malaria presenting as nonconvulsive status epilepticus are extremely rare. The case presented involves psychiatric symptoms, with the electroencephalogram indicated nonconvulsive status epilepticus associated with cerebral malaria.

**Case presentation:**

A 53-year-old male, was urgently admitted, due to confusion and abnormal behaviour for 10 h. The patient returned to China after developing a fever while working in Tanzania two months ago. The blood smear revealed *Plasmodium vivax* and *Plasmodium falciparum*, and he was diagnosed with malaria. He recovered following anti-malarial treatment. After admission, the patient was confused, unable to communicate normally, and unwilling to cooperate with the physical examination. *Plasmodium* was not found in the blood smear, but the DNA sequence of *P. falciparum* was discovered using metagenomic next-generation sequencing of cerebrospinal fluid. Brain MRI revealed no significant abnormalities. Continuous electroencephalogram monitoring revealed that the patient had non-convulsive status epilepticus, which was treated with diazepam and levetiracetam. The patient had normal consciousness and behaviour. He received anti-malarial treatment for two weeks and fully recovered.

**Conclusions:**

This case demonstrates that nonconvulsive status epilepticus can be a manifestation of cerebral malaria. It is imperative for attending physicians to heighten vigilance when encountering patients with a history of travel to malaria-endemic regions or a prior malaria infection, especially in the presence of unusual clinical presentations.

## Background

Although China has completely eliminated domestic malaria, there are still imported cases from endemic regions each year. Malaria is an infectious disease caused by *Plasmodium* parasites [1]. Parasites are transmitted by the bites of infected female mosquitos. The most common types of *Plasmodium* infections in humans are *Plasmodium falciparum* and *Plasmodium vivax* [1, 2]. Cerebral malaria (CM) is the most severe complication of *P. falciparum* malaria, characterized by high fever, coma, and seizures, often leading to a fatal outcome [3, 4]. The World Health Organization (WHO) defines cerebral malaria as a state of prolonged unconsciousness lasting over one hour, following the termination of seizures or correction of hypoglycaemia, with evidence of *P. falciparum* in the blood, after excluding other causes of encephalopathy [5]. This case reports a patient presenting primarily with confusion and abnormal behaviour, in whom electroencephalography (EEG) revealed nonconvulsive status epilepticus (NCSE) associated with cerebral malaria (CM), aimed at enhancing clinicians' awareness of this presentation.

## Case presentation

The patient, a 53-year-old male, was urgently admitted, due to confusion and abnormal behaviour for 10 h. Ten hours earlier, the patient was found sitting on the stairs by neighbours, exhibiting confusion and incoherent speech, but no limb movement impairment, vomiting, or convulsions. He was taken to the local hospital for a thorough examination, which included a head CT, CTA, and CTP, which revealed no significant abnormalities. With the exclusion of a history of cerebrovascular disease, suspicion arose regarding encephalitis, and symptomatic treatment, including anti-infection and fluid replacement to maintain internal homeostasis, was initiated. However, the patient did not improve, prompting family members to request that the patient be transferred to tertiary hospital for further diagnosis and treatment.

The patient's history revealed that he had a fever about two months ago, while working in Tanzania, and received three days of intravenous treatment. He returned to China after his fever had subsided. He developed intermittent fever and chills after one week, prompting a comprehensive examination in the local hospital, which confirmed the presence of malaria parasites (mixed infection of *P. vivax* and *P. falciparum*). He was admitted and started on regular medication (artemisinin-based combination therapy with artemether-lumefantrine and primaquine), which resulted in symptom relief. He was discharged because no malaria parasites were found in his blood. He took oral artemether-lumefantrine for three weeks, weekly blood smear revealed no evidence of malaria parasites, prompting him to discontinue anti-malarial medication.

When the patient was admitted, his body temperature was 38.1 °C, pulse was 90 times per minute, breathing rate was 19 times per minute, and blood pressure was 148/90 mmHg. He was disoriented with delayed reactions, bilaterally dilated pupils approximately 3 mm in diameter, responsive to light reflex, normal limb muscle tone. He was uncooperative with examination for bilateral anterior sensation, vibration sense, proprioception, and kinesthesia. Deep and superficial reflexes were normal, the Babinski sign was negative, and meningeal irritation sign was also negative. Blood malaria parasite testing was negative. White blood cell count was 11.03*10^9/L, the neutrophil percentage 81.7%, and the eosinophil percentage was 0.0%. Coagulation function, liver and kidney function, electrolytes, and procalcitonin levels were all normal. Lumbar puncture revealed an intracranial pressure of 150 mmH2O, and no significant abnormalities were found in cerebrospinal fluid examination (routine, biochemical, smear). Metagenomic next-generation sequencing of cerebrospinal fluid (CSF) revealed the DNA sequence of *P. falciparum*. The patient was diagnosed with cerebral malaria. Treatment included intravenous artemisinin 120 mg at 0, 12, and 24 h, followed by daily doses. The patient remains unconscious and incapable of communicating.

**Fig. 1:**
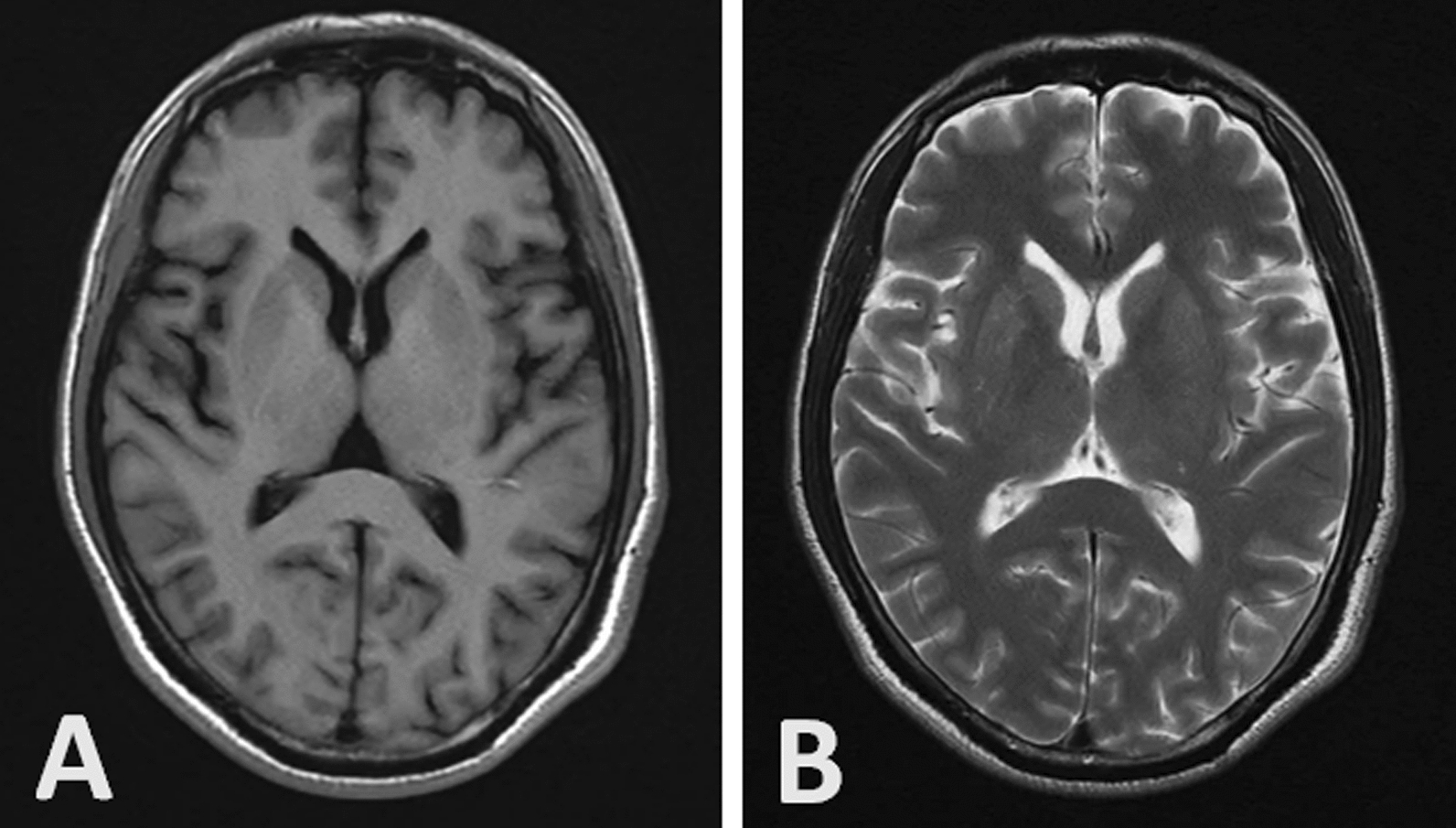
Head MRI of the patient shows no abnormalities in either the T1 (**A**) or T2 (**B**) sequence

**Fig. 2:**
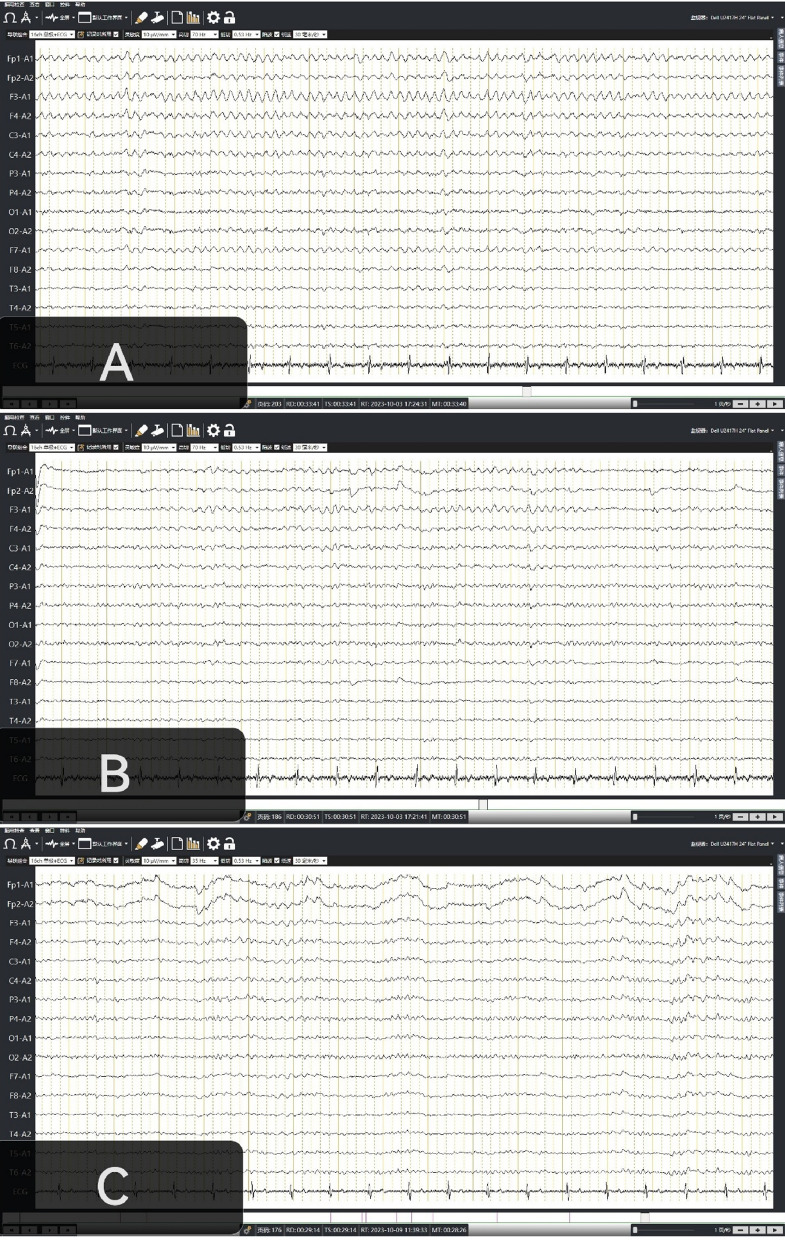
**A** When the patient was confusion, continuous video EEG monitoring revealed rhythmic theta waves (4–5 Hz) dominated in the frontal lobe; **B** After a slow intravenous administration of diazepam10 mg, the frequency of theta rhythm in the frontal lobe was increased while the amplitude decreased; **C** normal EEG before discharge

The results of brain MRI revealed no significant abnormalities (Fig. 1). The video EEG monitoring indicated more than 30 min continuous rhythmic waves with frequency of approximately 4–5 Hz (theta rhythm) in each one hour recording (Fig. 2A). The patient was suspected to have NCSE. The patient received a slow intravenous bolus of 10 mg diazepam. His consciousness was improved, and he could answer some simple questions. On the EEG, the frequency of θ rhythm in the frontal lobe becomes faster while the amplitude decreased (Fig. 2B). The patient was treated with levetiracetam 500 mg bid. His condition had gradually improved after one week, with progressive recovery of consciousness. Repeat video EEG revealed no nonconvulsive seizures (Fig. 2C). Malaria parasite was not detected in blood metagenomic next-generation sequencing. The patient was discharged after 2 weeks with a full recovery.

## Discussion

Malaria is an infectious disease caused by *Plasmodium* genus parasites, with *P. falciparum* and *P. vivax* responsible for the majority of human infections, particularly in Southeast Asian countries and India, where *P. vivax* is more prevalent(2). Severe malaria can cause anaemia, hypoglycaemia, metabolic acidosis, recurrent seizures, coma, or multiorgan failure, resulting in over 600,000 deaths annually [6].

Cerebral malaria is the most severe complication of *P. falciparum* malaria [3, 4]. Some perspectives suggest that CM is not a single condition but rather a collection of four distinct symptom clusters, including postictal state after prolonged seizures, NCSE, severe metabolic disturbances, and primary neurological syndromes [7]. Two potential mechanisms for CM pathogenesis have been proposed: vascular occlusion and the inflammatory hypothesis [8, 9]. CM has a mortality rate of up to 20%, and survivors face a high risk of long-term neurological sequelae, such as seizures, cognitive impairment, neurobehavioural sequelae, and focal neurological deficits [10]. The divergent outcomes of death and neurological sequelae may be attributed to different underlying mechanisms, with neurological sequelae linked to recurrent seizures and profound and prolonged coma, whereas death is associated with hypoglycaemia and acidosis [11].

NCSE is characterized by continuous epileptiform discharges on EEG, clinically presenting as a persistent state of nonconvulsive seizures. Clinical manifestations may include altered consciousness, aphasia, amnesia, delirium, and agitation, with occasional automatisms, nystagmus-like movements, and mild tremors in the face, mouth, abdomen, and limbs. The diagnostic criteria for NCSE are as follows: (1) Presence of sharp waves or sharp-slow wave complexes with an average frequency exceeding 2.5 Hz, with a minimum duration of a single episode being 10 min, or a cumulative duration exceeding 12 min within 1 h; (2) Gradual improvement in clinical symptoms and EEG patterns following intravenous administration of benzodiazepines and anti-epileptic drug therapy [12]. The International League Against Epilepsy (ILAE) has redefined NCSE and its classification, categorizing NCSE into four subtypes: absence status epilepticus, focal status epilepticus without impaired consciousness, focal status epilepticus with impaired consciousness, and coma in NCSE [13]. The mortality rate of NCSE due to epilepsy is approximately 3%, while that arising from an underlying disease is approximately 27%, the leading causes of death in NCSE are primarily related to underlying etiologies or subsequent complications and are rarely directly attributed to NCSE [14]. Continuous monitoring of the electroencephalogram is required for diagnosis of NCSE. This is especially true in malaria-endemic countries or regions with limited medical resources, where are lack of continuous EEG monitoring. This is probably the reason why clinicians know so little about rates of NCSE presentation with cerebral malaria.

It is worth noting that *P. falciparum* is a known seizure trigger, with an increased risk of seizures as parasitaemia rises [15]. Seizures and status epilepticus can occur during the active phase of intracranial infection in the majority of parasitic infections [16]. The current treatment for uncomplicated malaria is artemisinin-based combination therapy, whereas CM requires intravenous administration of the anti-malarial drug artesunate [17].

## Conclusion

This case demonstrates that NCSE can be a manifestation of CM. It is imperative for attending physicians to heighten vigilance when encountering patients with a history of travel to malaria-endemic regions or a prior malaria infection, especially in the presence of unusual clinical presentations.

## Data Availability

Not applicable.

## Abbreviations

CM: Cerebral malaria
CT: Computed tomography
CTA: Computed tomography angiography
MRI: Magnetic resonance imaging
EEG: Electroencephalogram
CSF: Cerebrospinal fluid
NCSE: Nonconvulsive status epilepticus
